# Induction and Analysis of the Alkaloid Mitragynine Content of a *Mitragyna speciosa* Suspension Culture System upon Elicitation and Precursor Feeding

**DOI:** 10.1155/2013/209434

**Published:** 2013-08-26

**Authors:** Nor Nahazima Mohamad Zuldin, Ikram Md. Said, Normah Mohd Noor, Zamri Zainal, Chew Jin Kiat, Ismanizan Ismail

**Affiliations:** ^1^School of Biosciences and Biotechnology, Faculty of Science and Technology, Universiti Kebangsaan Malaysia, 43600 Bangi, Selangor, Malaysia; ^2^School of Chemical Sciences and Food Technology, Faculty of Science and Technology, Universiti Kebangsaan Malaysia, 43600 Bangi, Selangor, Malaysia; ^3^Institute of System Biology, Universiti Kebangsaan Malaysia, Bangi 43600, Selangor, Malaysia

## Abstract

This study aimed to determine the effects of different concentrations and combinations of the phytohormones 2,4-dichlorophenoxy acetic acid (2,4-D), kinetin, 6-benzylaminopurine (BAP), and 1-naphthaleneacetic acid (NAA) on callus induction and to demonstrate the role of elicitors and exogenous precursors on the production of mitragynine in a *Mitragyna speciosa* suspension culture. The best callus induction was achieved from petiole explants cultured on WPM that was supplemented with 4 mg L^−1^ 2, 4-D (70.83%). Calli were transferred to liquid media and agitated on rotary shakers to establish *Mitragyna speciosa* cell suspension cultures. The optimum settled cell volume was achieved in the presence of WPM that contained 3 mg L^−1^ 2,4-D and 3% sucrose (9.47 ± 0.4667 mL). The treatment of cultures with different concentrations of yeast extract and salicylic acid for different inoculation periods revealed that the highest mitragynine content as determined by HPLC was achieved from the culture treated with 250 mg L^−1^ yeast extract (9.275 ± 0.082 mg L^−1^) that was harvested on day 6 of culturing; salicylic acid showed low mitragynine content in all concentrations used. Tryptophan and loganin were used as exogenous precursors; the highest level of mitragynine production was achieved in cultures treated with 3 **μ**M tryptophan and harvested at 6 days (13.226 ± 1.98 mg L^−1^).

## 1. Introduction 

*Mitragyna speciosa* is a medicinal tree in the Rubiaceae family that is native to Southeast Asia. It has been traditionally used in Thailand and Malaysia for its psychoactive properties; however, its use in these countries is now illegal. In Southeast Asia, the fresh leaves are commonly chewed, often continuously, by workers or manual laborers who seek its numbing, stimulatory effect. The leaves and bark of *M. speciosa *are used to cure opium addiction. These tissues contain many alkaloids, including mitragynine, mitraphylline, and 7-hydroxymitragynine; the latter is currently thought to be the most likely candidate for the primary active chemical in the plant. Mitragynine is the dominant alkaloid, and it has been assumed to be the physiologically active constituent that has morphine-like properties. It confers pain-threshold elevating and antitussive properties but lacks addictive properties [1].

Currently, the natural habitats of many plants are disappearing due to environmental and geopolitical instabilities; this loss of natural habitat for plants makes it difficult for human to acquire important secondary metabolites and prevents the discovery of many potentially useful compounds [2]. Important plant-derived drugs can still be obtained commercially by extracting the compounds from their whole-plant sources. The chemical synthesis of these compounds often results in the loss of their activity. The compounds contain highly complex structures with many chiral centers, and this complexity may contribute to their biological activities and to the difficulty in their economical synthesis [3]. Therefore, it is important to use a new alternative method to improve the content and productivity of the active ingredients in these plants. Plant tissue culture methods have been developed for many other endangered medicinal plants, such as *Curculigo orchioides* [4], *Podophyllum hexandrum* [5], *Hypoxis hemerocallidea* [6], and *Saussurea involucrata* [7]. Plant cell culture is considered to be a promising alternative for producing bioactive compounds that are difficult to be obtained by chemical synthesis or plant extraction [3]. Tissue culture is an attractive method because each plant cell culture exhibits totipotency, wherein the cell mass contains the full set of genes that are necessary for all of the plant functions, including secondary metabolism [8]. Cell culture systems are useful for the large-scale culture of plant cells, which produces a continuous and reliable source of secondary metabolites that can be purified easily due to the absence of significant levels of pigments. This approach avoids all seasonal plant growth constraints and eliminates geographic barriers for the production of secondary metabolites [9].

The major limitation in the production of secondary metabolites by plant cell culture technology is the low yield of secondary metabolites. The yield could be improved by standardizing the culture environment [10] and manipulating plant cell cultures to improve the production of target compounds by employing elicitors, abiotic stresses, and other approaches regardless of their mechanism [11]. The synthesis of target secondary metabolites in plant cell tissue cultures can be induced by applying physical, chemical, and biological elicitors. These elicitors mimic the effects of stresses and thereby activate the plant biochemical system; this induction results in the increased production of secondary metabolites in plant tissues [12]. The elevated production of desired products by elicitation has been reported in many studies, such as the production of indole alkaloid ajmalicinine from *Catharanthus roseus* cultures that were elicited by the fungi *Trichoderma viride* [13], rosmarinic acid and eugenol from *Ocimum basilicum* that was elicited by chitosan [14], beta-amyrin from *Medicago truncatula* that was elicited by a yeast elicitor [15], and taxol from *Taxus chinensis* [16] and ginsenoside from *Panax* ginseng that were elicited by methyl jasmonate [17]. It also has been reported that the addition of precursors or intermediate compounds involved at the beginning of the secondary metabolic biosynthetic pathway to the culture media sometimes stimulates the production of secondary metabolites [18]. Several attempts to induce or increase the production of plant secondary metabolites by supplying precursors or intermediate compounds have been performed, such as in the production of the alkaloid lunarine from *Lunaria annua* that was treated with phenylalanine [19], ajmalicine and strictosidine from *Catharanthus roseus* that was treated with secologanin, loganin, or loganic acid [20] and tryptophan [13], vanillin and capsaicin from *Capsicum frutescence* that was treated with the ferulic acid anvanyllyamine [21], anthocyanin from strawberry cultures that were treated with phenylalanine [22], and bilobalide and ginkgolides from *Ginkgo biloba* that was treated with terpenoid [23]. Therefore, the major objectives of this study were to establish an *M. speciosa* tissue culture system and to manipulate the culture environment and cell culture by elicitation and precursor feeding to increase the production of the alkaloid mitragynine.

## 2. Materials and Methods

### 2.1. Chemicals

Mitragynine standards were obtained from the School of Chemistry Sciences and Food Technology, Faculty of Science and Technology, UKM; WPM medium was obtained from Duchefa; *α*-naphthalene acetic acid (NAA), 2,4-dichlorophenoxy acetic acid (2,4-D), 6-benzylaminopurine (BAP), and kinetin were purchased from Sigma; yeast extract, salicylic acid, and tryptophan were purchased from Merck; and loganin was purchased from ChromaDex USA.

### 2.2. Plant Materials

*Mitragyna speciosa* plants were collected from Padang Siding, Perlis, and were grown at glass-house Biotechnology Laboratory, UKM. The petioles and young leaves were used as the explants in this experiment. The explants were washed thoroughly with running tap water for 30 min. The explants were then sterilized with 70% alcohol for 15 seconds and were washed three times. The explants were soaked for 30 min in 40% clorox bleach that contained three drops of Tween-20 and were then washed three times with distilled water. Next, the explants were dried on petri plates that contained a layer of filter paper. After trimming the cut size, the surface-sterilized explants were planted on the culture medium.

### 2.3. Callus Culture

In this study, woody plant medium (WPM) was supplemented with 3% sucrose as a carbon source. The WPM pH was then adjusted to pH 5.8 ± 0.1 with either 1 N HCl or KOH, and it was solidified with 0.7% agar before it was autoclaved at 121°C for 15 min. The different explants which is petiole and young leaves were cultured on various concentrations of 2,4-D (2, 4, 6, and 8 mg L^−1^) and were tested to determine which explants produced an optimal callus induction. WPM that was supplemented with 2,4-D, NAA, BAP, and kinetin individually or in combination at different concentrations was also used to study the effects of the different media components on callus induction. The cultures were incubated in a growth chamber at 25 ± 2°C. Each experiment had 24 replicates of explants and was repeated three times. The percentage of callus induction per plate was documented for up to 8 weeks. The culture medium that was not supplemented with any plant growth regulators was used as the control in this study.

### 2.4. Establishment of Suspension Culture

The establishment of the suspension culture was initiated by inoculating 2 g of finely chopped callus into 25 mL of liquid WPM in a 100 mL Erlenmeyer flask; it was incubated on a rotary shaker at 125 rpm. The cell suspension cultures were then maintained at 25 ± 2°C. Different concentrations of 2, 4-D (1 to 5 mg L^−1^) and sucrose (3 to 5% w/v) were tested to produce a rapid-growing and well-dispersed suspension culture of *Mitragyna speciosa*. Each treatment contained three replicates. The growth of the *Mitragyna speciosa* cell suspension culture was measured by the settled cell volume (SCV). The SCV was determined by allowing the suspension culture to sediment for 5 min in a sterile, graduated tube.

### 2.5. Preparation of Elicitors and Precursors

Filter sterilized elicitors and precursors were added to the subcultures of *M. speciosa* suspension cultures at varying concentrations. Yeast extract and salicylic acid were used as the elicitors at final concentrations of 100, 250, and 500 mg L^−1^, whereas tryptophan and loganin were used as the exogenous precursors at final concentration of 3, 6, and 9 *μ*M. These compounds were added to the *M. speciosa* suspension culture to test mitragynine production. Suspension culture medium that lacked elicitors and precursors was used as the control in this experiment. The cells were harvested six days and twelve days after treated with elicitor and exogenous precursor to analyze the mitragynine content.

### 2.6. Extraction

To extract the alkaloids, the cells were separated from the liquid media and ground with liquid nitrogen. Approximately 8 g was collected before it was extracted with 50 mL of methanol for three days. The methanol extract was filtered, and new methanol was added to the remaining cells; this process was repeated three times. The methanol filtrates were combined and evaporated under reduced pressure. The crude methanol extract was dissolved in a 10% acetic acid solution; it was shaken well and allowed to stand overnight. The acidic filtrate was adjusted to pH 11 with sodium carbonate and extracted with chloroform. The chloroform extract then was washed, dried over anhydrous sodium sulfate, and evaporated to yield a dry, crude alkaloid extract.

### 2.7. HPLC Quantification of Mitragynine

The presence of mitragynine in the samples was verified by the comparison of the Rt and UV spectral peaks of the sample with the standard peak. The HPLC system consisted of a Waters 2707 autosampler, a Waters 600 controller, and a Waters 2998 photodiode array detector. The data were collected and processed using the Empower Software System.

The analytical method was performed using Column *χ*Bridge C18 (size 5 *μ*m, 4.6 × 250 mm) from Waters. The mobile phase was a methanol-water mixture (80 : 20, v/v) and was filtered separately before it was mixed through a 0.22 *μ*m nylon membrane filter. The flow rate was 0.6 mL/min, and the autosampler vials were at an ambient temperature of 25 ± 1°C. A sampler volume of 10 *μ*L was injected, and the detector was set at 254 nm. A standard calibration curve was constructed by injecting different concentrations of mitragynine standard. The calibration curve for the mitragynine standard was linear (1 mg L^−1^–5 mg L^−1^) with a regression factor of 0.989240.

### 2.8. Statistical Analysis

Analysis of variance (ANOVA) was performed using the Statistical Analysis System (SAS) Version 9.0 software [24] to determine the significance of the treatment effects for each experiment. A *P* value of < 0.05 was considered to be significant.

## 3. Results

### 3.1. Plant Growth Regulator and Explants Effects on Callus Inductions

The petiole and leaf explants were placed on WPM that was supplemented with different concentrations of 2,4-D (2, 4, 6, 8, and 10 mg L^−1^) for callus induction. The optimum callus induction was obtained from leaf explants that were WPM supplemented with 4 mg L^−1^ of 2,4-D which is 20.83% (Table 1). For petiole explants, the optimum callus induction percentage was obtained with WPM that was supplemented with 4 mg L^−1^ of 2,4-D (70.83%) (Table 1). This concentration significantly affected the callus induction when compared to the control and the other concentrations of 2,4-D. Leaf explants produced a low efficiency of callus induction in all of the treatments compared to petiole explants. The callus cultures derived from the leaf explants grew quite slowly in all of the tested concentrations. No callus induction was observed in leaf explants cultured on WPM that was supplemented with 8 or 10 mg L^−1^ of 2,4-D. The leaf explants that did not induce callus turned brown and died. In general, media that contain high auxin and low cytokinin concentrations promote cell proliferation that results in callus formation [25]. To study the effects of plant growth regulators (PGRs) on callus culture, different PGRs were tested at different concentrations and in various combinations on the petiole explants. The combination of 2,4-D (2, 4, and 6 mg L^−1^) and kinetin (1 and 3 mg L^−1^) did not provide a responsive effect on the callus induction percentage compared to WPM that was supplemented with 2,4-D alone (Table 2). However, the combination of BAP and NAA showed that the optimum callus induction percentage was only achieved by 50% of callus induction on WPM containing 0.5 mg L^−1^ BAP + 4 mg L^−1^ NAA (Table 3). The callus that was obtained from the petiole explants that were cultured with 2,4-D and kinetin was friable and whitish in color, while the callus that was obtained from BAP and NAA was more compact, hard, and globular in shape. To establish the cell suspension cultures, the callus from the petiole explant that was cultured on WPM that was supplemented with 4 mg L^−1^ 2,4-D was elected to initiate the *M. speciosa* suspension cultures.

### 3.2. Establishment of Cell Suspension Culture

**Figure 1:**
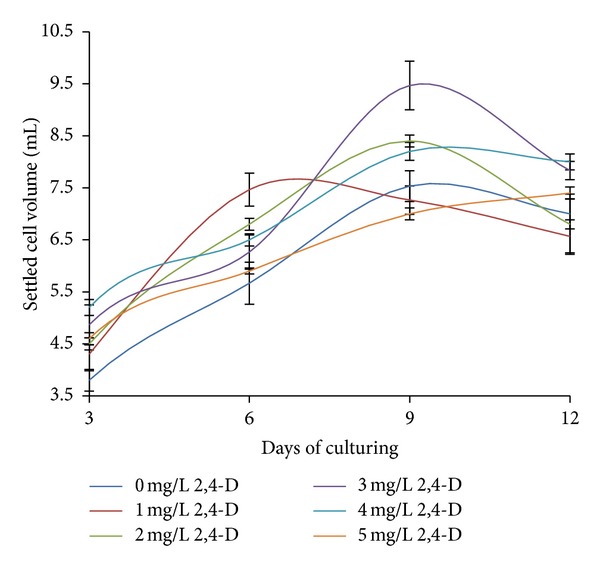
Effects of different concentrations of 2,4-D on the establishment of the *Mitragyna speciosa* cell suspension culture as measured by the settled cell volume (mL) in 25 mL of cell culture. The bars indicate the standard error.

To study the effects of auxin on the establishment of cell suspension culture, 2,4-D was used at concentrations from 1 to 5 mg L^−1^. As shown in Figure 1, 3 mg L^−1^ of 2,4-D was found to induce the best growth among all of the treatments, and it produced the highest measurements of SCV (9.47 ± 0.4667 mL). As the day progressed, the percentage of cell viability for most of the treatments decreased, and the color of the callus darkened. This may have been caused by the secretion of phenolic compounds, which may have led to the death of the heterogeneous suspension cultures after 9 days of culture. Concentrations of 2,4-D that were higher than 3 mg L^−1^ did not show improvement of the cell growth. The growth pattern for the 2, 3, and 4 mg L^−1^ treatments showed an inactive sigmoidal shape. This may have been caused by the adaptation of the suspension cells to the newly inoculated environment during the lag phase; as they entered the log phase, the cells eventually adapted to the new environment and started to grow exponentially by utilizing the nutrients that were provided [26]. In the 1 mg L^−1^ 2,4-D treatment, the cells initially grew rapidly, but the growth subsequently decreased drastically after 6 days of culture; conversely, at 5 mg L^−1^ of 2,4-D, a slow growth pattern was initially observed, and the culture continued to increase at 12 days of culture, while the other treatments decreased after 9 days of culture (Figure 1).

**Figure 2:**
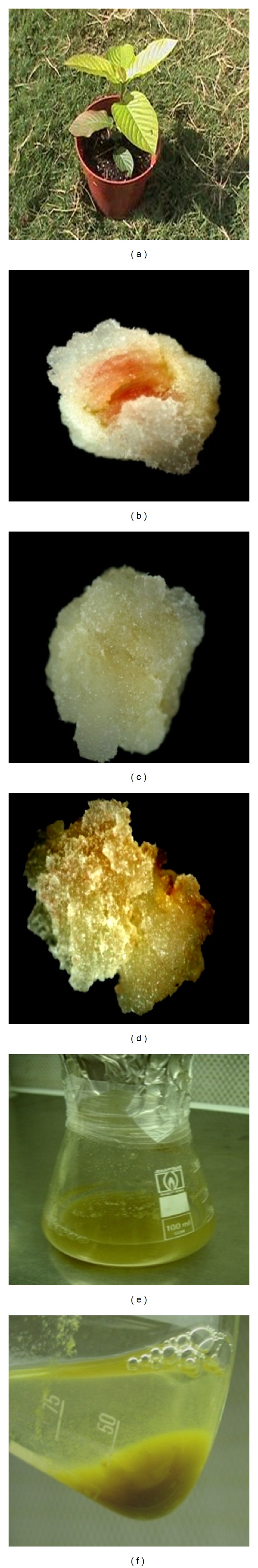
(a) *Mitragyna speciosa* tree, ((b)–(d)) callus of *Mitragyna speciosa*, and ((e)-(f)) *Mitragyna speciosa* suspension culture.

**Figure 3:**
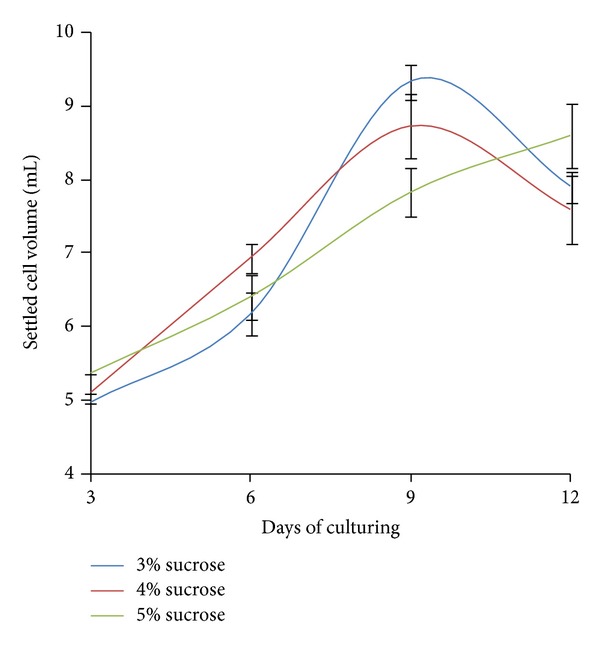
Effects of different sucrose concentrations of sucrose on the establishment of the *Mitragyna speciosa* cell suspension culture as measured by the settled cell volume (mL) in 25 mL of cell culture. The bars indicate the standard error.

In another experiment, the growth of the cell suspension culture was tested in the presence of 3 different concentrations of sucrose as a carbon source.  Figure 2 showed callus and suspension culture of *Mitragyna speciosa*. After 12 days of observation, the 3% (w/v) sucrose treatment facilitated optimum growth as measured by SCV (9.33 ± 0.219 mL) (Figure 3). However, although the cultures that were treated with 3% and 4% (w/v) sucrose decreased after 9 days of culture, the 5% (w/v) sucrose treatment caused no sudden decline in the cell-growth pattern. Although the 3% sucrose treatment provided the highest SCV, the culture initially grew slowly when compared to the other treatments; however, its growth suddenly increased on day 9. To achieve optimum cell growth, different plants require different carbon source concentrations due to the plants' different enzymatic metabolism [27]. Therefore, the sugar availability should initiate different responses that affect plant metabolism, growth, and development.

### 3.3. Elicitation

**Figure 4:**
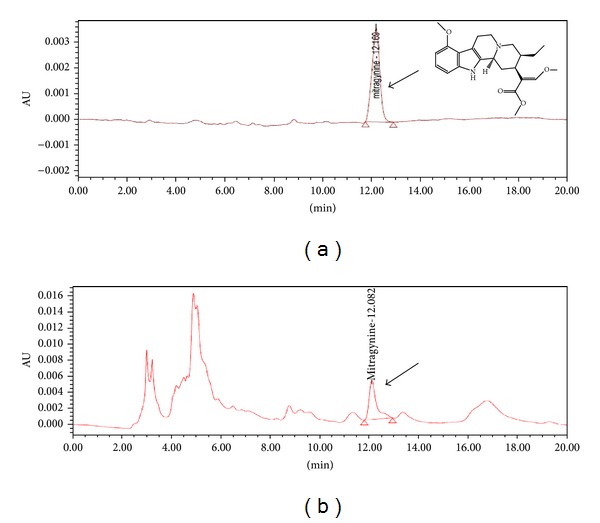
(a) HPLC chromatogram for the mitragynine standard and (b) chromatogram for the sample.

**Figure 5:**
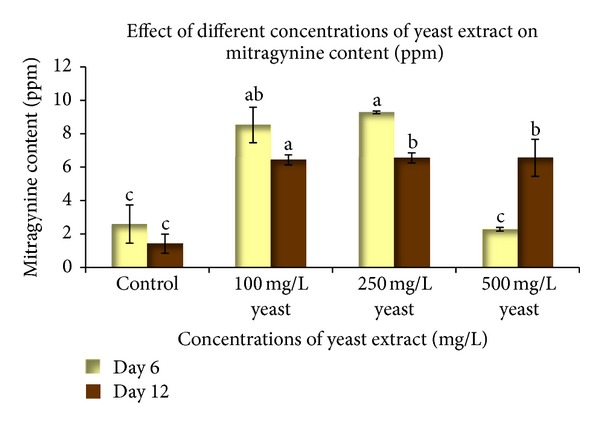
The effect of different yeast extract concentrations at different culture times on the mitragynine content in the *Mitragyna speciosa* suspension culture. The bars indicate standard error. *Values with the same letters are not significantly different according to Duncan's multiple range test.

**Figure 6:**
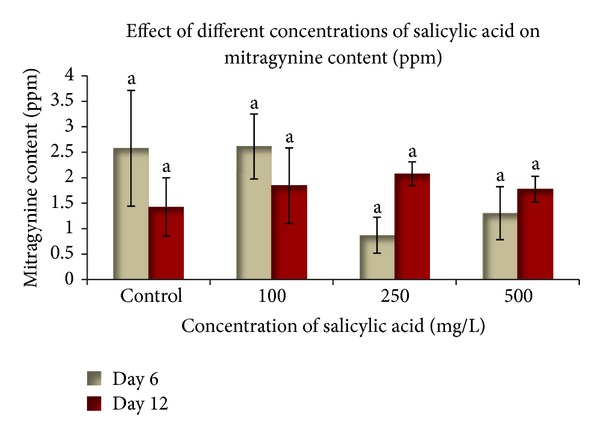
The effect of different salicylic acid concentrations at different culturing times on the mitragynine content in the *Mitragyna speciosa* suspension culture. The bars indicate standard error. *Values with the same letters are not significantly different according to Duncan's multiple range test.

To determine the effects of various concentrations of elicitors on mitragynine production, the cell suspension cultures were harvested six and twelve days after inoculation. A culture that lacked elicitors was used as the control. The mitragynine content was calculated using the calibration curve that was built with the Empower Software System (Figure 4). Different concentrations of yeast extract (100 mg L^−1^, 250 mg L^−1^, and 500 mg L^−1^) were added to a 100 mL Erlenmeyer flask that contained 25 mL of WPM.  Figure 5 shows the quantitative accumulation of mitragynine in response to different yeast extract concentrations at different harvesting times. The elicitation with yeast extract showed that the optimum mitragynine content was achieved with 250 mg L^−1^ yeast extract at six days of culture (9.275 ± 0.082 mg L^−1^). This value is significantly different than that of the control. However, the elicitation with 500 mg L^−1^ yeast extract provided the lowest mitragynine content at six days of inoculation. This demonstrates that the “overloading” of the elicitor can have adverse effects. The phase at which the plant cell suspension was harvested also affected the product accumulation. The cultures that were harvested at twelve days of culture showed no significant differences in the mitragynine content among all of the treatments. The inoculation with the yeast extract for six days of culture more effectively induced the mitragynine content when compared to twelve days of culture (Figure 5). These differences demonstrate that the biochemical changes that are associated with the induction of secondary metabolism following the addition of elicitors are very complex; it is likely that different metabolic systems can be affected, and there is a large amount of biochemical alteration [28]. In other experiments, the elicitation with all of the salicylic acid treatments did not significantly affect mitragynine production with respect to the control (Figure 6). This result showed that salicylic acid cannot stimulate mitragynine production. Unsuitable elicitor concentrations may cause unsuccessful elicitation, which indicates that a successful elicitation is a challenging process that requires intense standardization [13]. In this study, salicylic acid was not found to be a suitable elicitor for the increased production of secondary metabolites, while it may be a suppressor that led to the low biomass production. The induction of secondary metabolites by an elicitor may cause an increased metabolite production and a decreased cell mass [29]. 

### 3.4. Precursor Feeding

**Figure 7:**
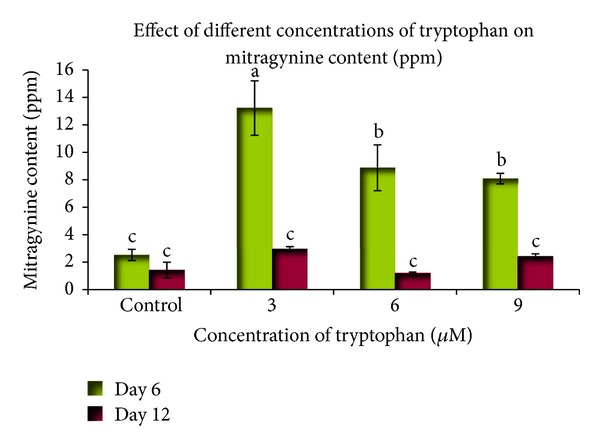
The effect of different tryptophan concentrations at different culture times on the mitragynine content in the suspension cultures of *Mitragyna speciosa*. The bars indicate standard error. *Values with the same letters are not significantly different according to Duncan's multiple range test.

**Figure 8:**
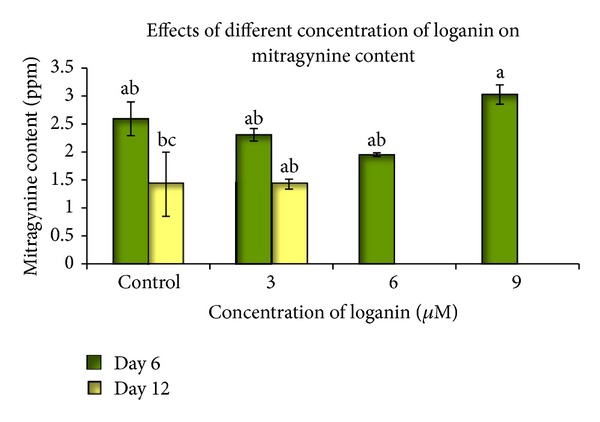
The effect of different loganin concentrations at different culture times on the mitragynine content in the suspension cultures of *Mitragyna speciosa*. The bars indicate standard error. *Values with the same letters are not significantly different according to Duncan's multiple range test.

The optimization of culture conditions, such as medium composition and the addition of biosynthetic precursors, may enhance the production of secondary metabolites, which may ordinarily be restricted by the lack of precursors or enhancers [30]. Two types of precursors, tryptophan and loganin, were used in this experiment. Different concentrations were tested at different inoculation time periods to study their effects on alkaloid accumulation. These precursors were added to 25 mL of WPM that contained an *M. speciosa* suspension culture. The HPLC analysis showed that the optimum mitragynine content was achieved following a 3 *μ*M tryptophan treatment at six days of culture (13.226 mg L^−1^). This value was significant when compared to other treatments (Figure 7). The comparison between the treatment periods showed that the precursor inoculation for six days produced a better response when compared to twelve days of culture. In another experiment, the highest mitragynine content was obtained from the 9 *μ*M loganin treatment that was harvested on day six of culture (3.028 mg L^−1^), although this value was not significantly different among the treatments (Figure 8). In most of the treatments, the mitragynine content was lower than in the control. No mitragynine was detected following the treatment with either 6 *μ*M or 9 *μ*M loganin at twelve days of culture. This experiment showed that loganin is not a positive inducer of mitragynine content in *M. speciosa* suspension culture. 

## 4. Discussion

The aims of this study were to develop a *M. speciosa* suspension culture system, to analyze the presence of mitragynine in the suspension cultures, and to use an elicitation and precursor feeding strategy to induce and increase mitragynine production in suspension culture. To obtain a good suspension culture, it is critical to initiate the suspension cultures from a friable callus source [31]. Callus is a dedifferentiated state of tissue that is achieved through the exogenous application of plant growth hormones *in vitro* [32]. Callus tissue is an essential material in plant cell culture systems. When a callus is introduced into a liquid medium and agitated, the cells disperse throughout the liquid to form a cell suspension culture. In theory, callus tissues are totipotent and have the potential to synthesize all of the compounds that are typically associated with an intact plant [33]. Therefore, an efficient procedure was developed for callus induction using different plant organs and different culture media that contained various hormone concentrations. 

The results showed that leaf explants induced callus formation at a lower level than the petiole explants. The petiole explants showed a high efficiency of callus induction; this may have occurred because the tissues were more responsive to the supplied hormones. The *in vitro* proliferation of tissues depends on the application of phytohormones and on the ability of the tissues to respond to these hormonal changes during the culture [34]. The slow callus induction of the leaf explant culture may have been caused by an unsuitable nutrient supply that was not compatible with the ability of the tissues to respond. Indra [35] suggested that the genotype, the environment, and the developmental stage of explants greatly influence their growth and are critical for the development of *in vitro* culture. Because the petiole showed a highly responsive callus production, petiole explants were used to test the effects of different types and concentrations of hormones on callus induction. 

The hormones that were used in this study were the combinations of 2,4-D and kinetin and BAP and NAA. The 2,4-D treatment alone without kinetin was found to have a higher organogenic potential and produced a loose friable, whitish-colored callus. The best results for callus proliferation and growth were achieved in a medium that contained 4.0 mg L^−1^ 2,4-D. As the friability of the cells increases, it is easier to achieve the full separation of the cells; this is ideal for the initiation of cell suspension cultures. Plant cell cultures are normally established and maintained on media that contain an auxin and a cytokinin. The removal of either hormone from the medium can result in the low induction of callus. This was observed in this study when WPM that lacked hormones (control) produced a low percentage of callus induction when compared to that with hormones. At the cellular level, auxins control basic processes, such as cell division and cell elongation. Because they can initiate cell division, they are involved in the formation of meristems that produce either unorganized tissue or defined organs. Cells that respond to auxin revert to a dedifferentiated state and begin to divide. To induce callus growth from the explants of dicotyledonous plants, a cytokinin is usually added to the medium in addition to an auxin. A few studies have reported successful results from using kinetin in combination with 2,4-D to induce callus formation; for example, a combination of 0.5 mg L^−1^ 2,4-D and 0.05 mg L^−1^ kinetin was used to induce callus from *Arabidopsis thali*ana [36], 0.1 mg L^−1^ kinetin and 0.5 mg L^−1^ 2,4-D induced callus in *Beta vulgaris* [37], and 5 mg/L 2,4-D and 5 mg/L kinetin induced callus in *Rudbeckia hirta* [38]. In the current study, kinetin, which is a cytokinin hormone, was used alone and in combination with auxin (2,4-D), but it did not stimulate a high percentage of callus induction when compared to 2,4-D alone (Table 2). This result indicates that kinetin cannot promote callus growth for *M. speciosa*. The application of 4 mg L^−1^ of the growth regulator 2,4-D is required to induce callus growth from petiole explants; however, kinetin did not support the role of 2,4-D to promote optimal callus growth. In another experiment, another combination of auxin and cytokinin (NAA combined with BAP) was tested on the promotion of callus proliferation. The result showed that 4 mg L^−1^ NAA that was combined with 0.5 mg L^−1^ BAP provided an optimum level of callus induction (50%) (Table 3). Using the Duncan multiple range test (DMRT), this result was significant with respect to the other treatments. The application of BAP or NAA alone did not promote callogenesis in the petiole segments in the *M. speciosa* cultures. The callus structure that was obtained from this experiment showed a globular shape with a white color, but the structure was hard. A hard and crusty callus structure is not suitable for the initiation of suspension cultures because it has a low dispersion and separation properties, and it is difficult to obtain single cells from a clump of cells. Therefore, the callus that was obtained from WPM that contained 4 mg L^−1^ 2,4-D was used to initiate *M. speciosa* suspension cultures. To prevent the callus cells from developing further, a cell suspension culture was established in which cell multiplication was the primary focus. The transfer of callus pieces into flasks that contain liquid media initiates the cell suspensions, in which the growth rates of the suspension-cultured cells in liquid media are generally higher than the growth rates on solid media. The liquid WPM that contained 3% sucrose and different concentrations of the plant growth regulator 2,4-D (1 to 5 mg L^−1^) was tested to determine the best culture medium for establishing the suspension culture. The suspension was placed on a rotary shaker to provide aeration and to uniformly disperse the cells in the suspension. A culture that consisted of a high percentage of single cells and a high settled cell volume was considered to be a good suspension. After initiating the cell suspension with 2 g of initial inoculum, the cell volume slowly increased over the course of 3–6 days and eventually increased rapidly after 6 days of culture. After 9 days, the volume of cells gradually began to decrease. Immediately after they are cultured, cell division resumes after a lag phase, which leads to the exponential or logarithmic growth phase; during this phase, there is a biomass increase. Observations from the growth study showed that these cell suspensions can be maintained by performing sequential subcultures during the early stationary phase at a time when cell aggregation is maximal to avoid genetic variation in the suspension culture and to obtain a homogeneous population. Based on the cell behavior in the suspension culture that was grown in WPM containing 3 mg L^−1^ 2,4-D, the stage of subculturing was identified to be within 6–9 days; this was considered to be the critical subculturing stage to obtain the maximum production of secondary metabolites. Because all of the treatments showed a declining stage after 9 days of culture, the nutrients in the medium may have been exhausted and/or toxic metabolic byproducts may have been formed. Using liquid WPM that contained the best 2,4-D level and 3% sucrose, two additional percentages of sucrose (4 and 5%) were tested with regard to the cell viability percentage and settled cell volume as in the previous experiment. Sucrose is the primary energy source for *in vitro* plant tissue cultures because they have insufficient autotrophic abilities [39]. Sucrose (2–5%) is the most popular carbohydrate that is used in tissue culture. In general, most tissue culture studies are performed using sucrose as the sole carbon source due to its efficient uptake across the plasma membrane [40]. Sucrose acts as an external energy source and contributes to the osmotic potential of the medium, which facilitates the absorption of mineral nutrients that are present in the medium and are essential for cell growth; therefore, the optimal osmotic pressure is required for optimal proliferation [41]. 

A significant effect of the carbon source concentration on culture growth has been reported in many studies, such as those in rice [42], patchouli [40], *Gentiana kurroo* [43], olive [44], and *Melastoma malabathricum* [45]. In the current study, 3% sucrose was used as a control. The growth analysis status showed that the optimal sucrose level to obtain good suspension cultures is 30 gL^−1^ (3%). This result indicated that sucrose levels that were higher than 3% gave low efficiency of cell suspension volumes. The high sucrose content reduced the water content in the cultured cells, which may explain why the higher sucrose levels showed a slow cell growth trend in *M. speciosa*. This can be explained because the higher concentration of sucrose in the culture medium can reduce or slow the cell biomass due by increasing osmotic potential, which subsequently reduces nutrient uptake. The optimal liquid medium (3 mg L^−1^ 2,4-D and 3% sucrose) for the suspension cultures was then used as a basal medium in the elicitation and precursor feeding studies. 

Two types of elicitors were used, yeast extract and salicylic acid, and two precursors were used, loganin and tryptophan. In this study, the optimum mitragynine concentration was achieved from the elicitation with 250 mg L^−1^ yeast extract in the cultures that were harvested at 6 days of culture (9.275 ± 0.082 mg L^−1^). This concentration was 1.5-fold higher than in the cells that were harvested at 12 days of culture. However, the result was not significantly different when compared to the treatment with 100 mg L^−1^ yeast extract, which produced 8.523 mg L^−1^. Both of the treatments were significantly different from the 500 mg L^−1^ yeast extract treatment. This result showed that at low concentrations of yeast extract higher concentrations of mitragynine were produced and at higher concentrations of yeast extract the mitragynine content was low. The type and concentration of the elicitor are critical to the elicitation process. A high elicitor dosage may induce a hypersensitive that can lead to cell death, whereas an optimum level is required for induction [13]. This statement was supported by our results, in which 500 mg L^−1^ yeast extract produced a low mitragynine level. Reports have also described the use of different concentrations of yeast extract, such as the elicitation of sesquiterpenes in *Nicotiana tabacum* [46], the elicitation of sanguinarine in *Eschscholtzia californica* [47], the accumulation of alkaloids in *Eschscholtzia californica* suspension cultures [48], secondary metabolism in *Astragalus chrysochlorus* cell cultures [49], and the production of plumbagin in *Plumbago rosea* L. suspension cultures [50]. Furthermore, the elicitation of mitragynine using salicylic acid resulted in a low mitragynine concentration for all of the treatments, which was also generally lower than that in the control. Therefore, salicylic acid is not a good elicitor for elevating the production of mitragynine. Some reports have also shown unsuccessful results using salicylic acid as an elicitor, such as tropane alkaloid production in *Atropa belladonna *[51] and the production of taxol in suspension cultures of *Taxus chinensis* which was increased more significantly by methyl jasmonate than by salicylic acid [52]. 

Our precursor study showed that an optimum mitragynine level was achieved following a six-day 3 *μ*M tryptophan treatment (13.226 mg L^−1^); the level was 5-fold higher than the control. This result is significantly different among all of the treatments. Low mitragynine concentrations were observed in the cell suspension cultures that were harvested at 12 days of culture when compared to those that were harvested at 6 days. Higher tryptophan doses (6 and 9 *μ*M) reduced the mitragynine concentration when compared to the 3 *μ*M tryptophan dose. Although the specific process of mitragynine biosynthesis is still poorly understood, mitragynine is known to belong to the monoterpene indole alkaloid (MIA) group. Strictosidine is a common MIA that gives rise to more than 2,000 specific MIAs in different plant species [53]. Tryptophan is the starting material in the MIA biosynthetic pathway and is converted to tryptamine by the tryptophan decarboxylase enzyme. Tryptamine combined with secologanin by the presence of the strictosidine synthetase enzyme and yields strictosidine. Various enzymatic conversion reactions lead to the synthesis of various compounds from strictosidine [54]. To enhance the secondary metabolite production in cultured cells, it is possible to feed the precursor or one of the intermediates into the metabolic pathway so that it may be subsequently converted into a final product [55]. Therefore, the loganin precursor was also studied to determine its effect on mitragynine production. However, loganin did not positively affect mitragynine production (Figure 8), whereas the cell suspensions that were treated with 6 or 9 *μ*M loganin that were harvested on day 12 did not produce any mitragynine. Therefore, instead of enhancing secondary metabolite production, loganin acted as an inhibitor. This may have resulted in low conversion of strictosidine from loganin and a secologanin. 

Based on the limited knowledge of mitragynine biosynthesis, the trial and error method were used to improve the secondary metabolite content in a *Mitragyna speciosa* suspension culture. Nevertheless, these preliminary studies provided data regarding the construction of a tissue culture system, and the elicitation study in the *Mitragyna speciosa* suspension culture can be used as a guideline to provide a more appropriate protocol for further studies of this species.

## Figures and Tables

**Table 1:** Effects of different concentrations of 2,4-D on the percentage of callus induction between leaf explants and petiole explants.

Concentration of 2,4-D (mgL^−1^)	Percentage of leaf explants that produced callus (%)	Percentage of petiole explants that produced callus (%)
0	4.167^bc^	9.72^d^
2	16.67^ab^	36.11^b^
4	20.83^a^	70.83^a^
6	4.167^bc^	26.39^bc^
8	0^c^	19.44^cd^
10	0^c^	19.44^cd^

Percentages within a column that have the same letter are not statistically significantly different according to Duncan's multiple range test at *P* ≤ 0.05.

**Table 2:** Effects of the combination 2,4-D and kinetin at different concentrations on the percentage of callus induction from petiole explants.

Treatments	Percentage of explants that produced callus (%)			
	2,4-D (mgL^−1^)			
	0	2	4	6
Kinetin (mgL^−1^)				
0	12.5^d^	37.68^b^	72.22^a^	27.77^bc^
1	26.387^bc^	4.167^d^	12.5^d^	25^c^
3	4.167^d^	31.92^bc^	36.11^bc^	36.11^bc^

Percentages within a column that have the same letter are not statistically significantly different according to Duncan's multiple range test at *P* ≤ 0.05.

**Table 3:** Effects of the combination NAA and BAP at different concentrations on the percentage of callus induction from petiole explants.

	Percentage of explants that produced callus (%)				
Treatments	NAA (mgL^−1^)				
	0	2	4	6	8
BAP (mgL^−1^)					
0	6.943^ef^	8.33^def^	37.5^b^	24.997^c^	20.833^cd^
0.5	4.167^f^	19.443^cde^	50^a^	12.5^def^	8.33^def^

Percentages within a column that have the same letter are not statistically significantly different according to Duncan's multiple range test at *P* ≤ 0.05.

## Abbreviations

WPM: woody plant medium
2,4-D: 2,4-Dichlorophenoxy acetic acid
BAP: 6-Benzylaminopurine
NAA: 1-Naphthaleneacetic acid.
